# Closed-Loop Neuroprosthetics: Advancing Long-Term Solutions for Brain-Level Function Restoration

**DOI:** 10.7759/cureus.116352

**Published:** 2026-09-16

**Authors:** Chetna Malga, Austin J Scholp

**Affiliations:** 1 Biology, Jordan High School, Katy, USA; 2 Biomedical Engineering, University of Iowa, Iowa City, USA

**Keywords:** biosensor, computational, neural implant, neuroprosthetic, recoverable conductors

## Abstract

Closed-loop neuroprosthetic devices use neural sensing coupled to real-time computational processing, feedback, and output applications to restore lost motor, sensory, or communication abilities. Despite having great potential, these devices have yet to make it into clinical practice due to unstable interfaces with the nervous system, where material fatigue, electrode degradation, inflammation, electrical drift, and other factors emerge over time to inhibit reliable sensor feedback and stimulation. This paper explores how these challenges grow exponentially at the cerebral level, where soft tissue, dense circuitry, and stochastic neuronal firing create significant hurdles to long-term implantation and recalibration. The author suggests that two lines of device development are slowly coming together: self-healing, deformable, and compliant interface materials that aim to maintain electrical coupling and adaptive control architectures that adjust decoding and feedback as biological conditions shift over time. By establishing the current neuroprosthetic implementations, persistent engineering challenges, and new design approaches, this paper presents a framework for neuroprosthetic devices that restore responsive function rather than provide short-term substitution.

## Introduction and background

A malfunction in a neuroprosthetic system is not simply an inconvenience; it can mean the return of paralysis, the loss of sensation, or the severing of a laboriously created route for thought transmission to other people and for everyday interactions. For individuals suffering from amputation, spinal cord injuries, strokes, or neurodegenerative diseases, these devices offer a path back to agency, but this path is tenuous if implanted interfaces do not have a long lifespan in vivo.

The populations concerned are substantial. Roughly 12 million people worldwide sustain a stroke each year, and over 100 million are living with its effects [1]. An estimated 250,000 to 500,000 people sustain a spinal cord injury (SCI) annually [2], with approximately 300,000 people living with SCI in the United States alone [3]. Limb loss affects around 1.6 million people in the United States, a figure projected to double by 2050 [4]. Even if only a fraction of these individuals are candidates for implanted interfaces, the scale of potential benefit is considerable.

Prosthetic systems have a long history, starting with mechanical limb replacements, implantable stimulators, and early brain-computer interfaces, but their clinical impact has been limited by our inability to make them function continuously and reliably because of biological material behavior and degradation. Closed-loop systems represent a departure from previously passive prosthetic replacements toward active monitoring of neuromuscular signals, continuous computation, and real-time output of required activation levels. Closed-loop systems continuously sense neural activity, process that information in real time, and provide feedback or stimulation in response. This paper hypothesizes that, at the brain level, true restoration requires durable, self-healing interface materials integrated with an adaptive control scheme; we define these neuroprosthetic systems, discuss their current applications, outline the remaining obstacles to permanence, and consider potential solutions.

Integrated design as a novel framework

Historically, neuroprosthetic development has proceeded along two largely independent tracks: materials engineers optimize electrode stability and biocompatibility, while control researchers develop decoding algorithms and feedback strategies. This sequential model assumes that robust hardware can be paired with adaptive software after the fact. However, this paper argues for a fundamental departure from this separation. Long-term restoration requires co-designed platforms where materials science and adaptive control architecture develop in parallel, each informing the other from the outset. Self-healing interfaces that reduce mechanical stress only realize their clinical benefit when paired with adaptive decoders that compensate for residual drift. Conversely, sophisticated algorithms cannot overcome hardware failures or signal degradation caused by material incompatibility. The framework presented here treats this integration not as an optimization preference but as a clinical necessity for devices intended to function reliably outside the laboratory.

Methods

Literature was identified through searches of PubMed and Google Scholar using key terms, including “closed-loop neuroprosthetics,” “neural interfaces,” “brain-computer interfaces,” “electrode degradation,” “adaptive control,” and “neural signal drift.” This narrative review prioritizes peer-reviewed primary research and review articles published between approximately 2010 and 2026, with emphasis on recent literature (2020-2026) while retaining foundational studies. Studies were selected for their relevance to electrode-tissue interactions, signal instability mechanisms, adaptive decoding strategies, and clinical applications in motor, sensory, and communication restoration. The scope reflects a focus on chronic brain-level implantation and long-term clinical translation of closed-loop neuroprosthetic systems.

## Review

Overview of the problem and application

Closed-loop neuroprostheses are implanted or body-coupled systems that continuously sense neural or physiological activity, perform computational operations on the sensed signals, and respond with altered stimulation or device behavior based on the detected state [5]. A critical distinction emerges between systems designed merely to resist degradation and systems designed to recover performance despite inevitable degradation. Performance-recoverable neuroprosthetics represent this latter approach. They are engineered from inception with the expectation that biological drift, material fatigue, and signal instability will occur, and they incorporate mechanisms (both material and algorithmic) to maintain function through these changes rather than prevent them entirely. This recovery-centered philosophy provides the organizing principle for this review and represents a novel conceptual contribution to how we approach long-term neuroprosthetic restoration. Closed-loop devices differ from user-controlled open-loop devices that deliver fixed outputs; closed-loop devices rely on continuous sensing, a feedback loop, and a real-time response so that control is physically and cognitively coupled to the user's current intention and state. This is critical because neural signals change over time due to learning, fatigue, and drift at the tissue-electrode interface, and the neuroprosthesis must adapt with the user rather than function as a static partner [6]. Functionally, the loop connects measurement, decision-making, and actions. The latter can include updating a motor decoder, changing stimulation parameters, or correcting modulation errors during device use. For this reason, closed-loop neuroprostheses are best viewed as adaptive therapeutic systems whose goal is to restore functionality through regulation and adaptation rather than acting as a fixed prosthetic replacement for the impaired structure.

Furthermore, the value of closed-loop neuroprosthetics is clearest when restoration is understood not as device performance alone but as the recovery of everyday agency across motor, sensory, and communication functions. In the motor domain, the ability to reach, grasp, or control movement can reduce dependence on caregivers and reopen work, self-care, and social participation, making decoding accuracy matter because it supports durable clinical function. Sensory restoration carries similar weight, since the return of touch, pressure, or partial vision can improve safety, embodiment, and confidence, while bidirectional systems are being developed specifically as safe, compact, and reliable tools for clinical use [7]. Restoration of communication is equally consequential because preserving speech or other expressive channels helps patients remain present in decisions, relationships, and public life. Across all three domains, long-term reliability is therefore a clinical requirement rather than an engineering preference, especially as implantable bioelectronic systems are increasingly expected to support chronic management where patient safety and system reliability are central [8]. In that setting, device failure means losing not just technical output but access to action, perception, and expression.

From a clinical standpoint, the limitations of implantable prosthetics become more evident over extended use, requiring evaluation months or years after implantation rather than at the time of implantation. Frequent micromotion, mechanical loading, and corrosion or insulation failure alter the electrode characteristics, while inflammation and fibrotic encapsulation alter and possibly weaken the tissue environment for recording and stimulation. This process is persistent across the central and peripheral interfaces and often results in drift and instability in recording accuracy, which erodes the device-level integrity that is necessary for reliable long-term operation for decoding and feedback control [9]. Similarly, chronic immune response and interface integration in peripheral nerve applications manifest as drift in the recorded signal, resulting in more frequent recalibration and reduced confidence in perceived sensory or motor output during normal use [10]. Unfortunately for closed-loop prosthetics, these degenerative mechanisms are not merely maintenance concerns but rather direct compromises of timing, accuracy, and the ability to trust the operation. The ability to endure within living tissues and, therefore, remain stable and functional is a foundational requirement for any design intended to operate continuously away from the clinical environment.

As demonstrated by studies with intracortical brain-computer interfaces, closed-loop design adds the most value when it is engaged directly during use as opposed to open-loop operation followed by separate calibration and reconstruction. Decoded neural control signals differ when the user is in closed-loop operation with feedback and develop over time with changing task needs and signal characteristics. Decoders that learn adaptively during this closed-loop operation will better represent the user’s intent (temporal domain considerations) than decoders that were pretrained before use [6]. In addition, drift is an inevitable process that reduces the effectiveness of steady-state control, and a model that was built with out-of-use training data may no longer be fit for purpose as control becomes more remote from the desired state. Closed-loop recalibration improves long-term performance both by raising baseline accuracy and by restoring control after drift-induced instability, without pausing the session for manual recalibration [11]. Viewed from this perspective, the benefits of adaptive brain-machine control are not just online with improved fidelity, but they also reflect resilience when neural control becomes more uncertain with time. As a result, continuous recalibration within the closed loop is clearly essential if everyday neuroprosthetic usage is to become a reality.

Motor Restoration

Motor restoration further illustrates the possibility of closed-loop neuroprostheses using the intent that remains available to individuals with paralysis and converting this into a command that allows movement. The closed-loop neuroprosthesis decodes cortical or peripheral signals into movement commands. These commands drive either an external device, such as a robotic arm or cursor, or the user’s own muscles through electrical stimulation. During the attempted movement, the system uses kinematic, muscular, or task-performance feedback to adjust stimulation and control parameters so that the output stays aligned with the user’s goal. In cases where the descending motor pathways from the cortex to the spinal cord are damaged, as in spinal cord injury or stroke, but voluntary motor plans can still be decoded, this closed-loop control is vital, as the final output can be taken from an external device or from function in the electrically activated muscles. In SCI, it has been shown that adaptive algorithms can use ongoing information about the movements to avoid false positives and stabilize gait detection and correction, allowing immediate control and long-term reorganization of functions rather than using triggering mechanisms alone [12]. Chronic fully implantable devices support this approach by enabling concurrent sensing and stimulation in a single implanted system. These devices have the potential to make complete continuous motor calibration possible while the user performs real functional tasks, rather than only during structured calibration sessions [13]. This sort of system brings motor restoration closer to real voluntary control that is simultaneously updated as the neural state and bodily outputs change.

Sensory Restoration

Similarly, sensory restoration is best understood as a bidirectional control problem in which the prosthetic system must both read the user’s intent and return tactile information quickly enough to shape the next action. Contact, pressure, and slip detected at an artificial limb or skin-coupled surface are converted into patterned stimulation so that grip force and hand position can be corrected during ongoing movement rather than after visual inspection alone. The goal is not simply to evoke sensation but to close the loop between action and perception through interfaces designed for feedback-controlled neuroprosthetics at the peripheral nerve level [10]. That requirement makes material compliance central, because implantable neural interfaces and wearable tactile systems work better when they conform to tissue mechanics while maintaining stable signal transmission over time [7]. In this sense, sensory restoration follows the same adaptive logic as motor control while confronting the harder task of reconstructing usable perception in real time.

Communication Restoration

Just as communication restoration generalizes closed-loop neuroprosthetics to the domain of language, its mission to remake intended combinations of speech elements into intelligible, timely dispatches for people in whom a neural or physical insult has cut off the normal speech pathway also hinges on this consideration. Practical efficacy here is profoundly contingent on low-latency sensing and decoding; delays between activity in the target neural site, system output, and user feedback can instantaneously destroy a turn-taking conversational exchange and cause one to lose access to control over the process [14]. A communication system cannot rely on a static dictionary or signal-set-to-term/segment/gesture correlation. Long-term recordings either drift or the individual changes over time; active decoders must be updated to adapt to shifting neural and behavioral input correlates [15]. Feedback is not an extra feature added to the system; it is part of the control loop that allows users to discover mistakes, to clarify the current output, and to stabilize performance over time and repeated use. Classification of a language state in isolation is thus of no utility; there must be the understanding that the output of any system must facilitate the practical business of communication: it must be intelligible, context-appropriate, responsive, and individual for the user. Speech neuroprosthesis has demonstrated why the business of restoration of communication is far from independent; even where it might seem that there are unique demands made upon the timing and usability of a neuroprosthesis, the demands of its adaptive architecture mirror those found in other domains of functioning in which the neuroprosthetics are applied.

Long-term brain-level restoration also depends on real-time adjustment, because the neural environment after implantation keeps shifting with learning, fatigue, tissue response, and ordinary changes in cognitive state. A decoder or stimulation policy set once can therefore drift out of alignment with the signals it is meant to interpret, weakening control and making feedback less dependable in daily use. Adaptive closed-loop systems are designed to counter that instability by updating control in response to changing neural signals and behavioral performance, an approach used to stabilize neuroprosthetic and rehabilitation-oriented brain-computer interfaces over time [16]. This is especially important in implanted systems, where long-term recording drift and user adaptation make static calibration inadequate for high-dimensional, nonstationary brain activity [15]. Real-time adjustment thus acts as the computational partner to durable hardware, translating biological change into continued function while underscoring the need for materials that can preserve the interface those algorithms rely on.

Technological challenges

The central material bottleneck in long-term brain implants is the electrode-tissue interface, where a device must continue to conduct, insulate, and adhere while embedded in tissue far softer and more mobile than the implant itself. Even small brain movements from pulsation, respiration, and head motion can concentrate stress at that boundary, and the frequency of micromotion strongly shapes the mechanical strain imposed on surrounding tissue and the implant surface [17]. That persistent mismatch does not remain purely mechanical. Inflammation and fibrous encapsulation around the electrode gradually insulate it from surrounding neurons, raising the impedance of the electrode-tissue interface and weakening the recorded signals [18]. As impedance increases and the interface thickens, stable sensing and stimulation become harder to sustain over chronic use. For that reason, long-term reliability depends less on making electrodes simply smaller or stronger than on designing them to mechanically match brain tissue. Materials that better conform to neural motion are therefore essential to preserving both electrical continuity and tissue compatibility over time.

Electrode-Tissue Interface Dynamics

Chronic neural implant loss due to mechanical fatigue occurs not from any high-deformation events but due to continued micromotion that occurs with each pulse, breath, and normal activity for a rigid interface within the soft tissue environment of the brain. This continued cycle of deformation can compromise coatings, crack thin wires, and cause a change in interface geometry, causing corrosion and changes in impedance that increase and destabilize current delivery and recording [19]. This same motion promotes inflammatory loading at the interface, which increases tissue encapsulation surrounding the interface. Encapsulation leads to corrosion and increased impedance that reduces the signal and performance as a result of a coupling of the mechanical and biological failures [20]. The electrochemical processes underlying electrode degradation vary with material composition and environmental conditions. For platinum electrodes, galvanic corrosion can occur when multiple metals are present, creating a potential gradient that drives oxidation of more reactive elements. Hydrolytic breakdown affects insulating polymers such as parylene and polyimide, where water penetration at microcracks causes bond cleavage and progressive loss of insulation integrity [18]. Iridium oxide electrodes, despite their superior charge injection capacity, are susceptible to potential-dependent oxidation when subjected to repeated stimulation pulses, causing irreversible transformation to nonconductive iridium hydroxide. Conducting polymers such as poly(3,4-ethylenedioxythiophene) (PEDOT) face dedoping and structural degradation with repeated cycling, reducing conductivity over time [18]. Each material class thus exhibits distinct failure modes that require tailored design interventions [18]. Platinum benefits from surface passivation and geometric redundancy to distribute current [18]. Iridium oxide requires potential limits and pulse protocols to maintain oxidation state stability. Conducting polymers benefit from cross-linking and structural reinforcement to resist dedoping [18]. Recognition of these specific electrochemical mechanisms allows the design of electrode materials and stimulation strategies that actively resist rather than merely tolerate degradation. As such, there is a growing role in implant design to increase compliance as a method to mitigate fatigue rather than as a material composition preference. Softer substrates, load-dispersing geometries, and soft interfaces can reduce mechanical mismatch and accommodate tissue movement at the edge of the interfaces. By reducing the mechanical mismatch and local injury, these devices can reduce the effects of fatigue to ensure chronic performance.

Chronic Inflammation and Glial Encapsulation

However, risks to feedback control neuroprostheses also occur in a more gradual manner through changes in signal characteristics that proceed even when electrodes remain physically intact. Signal drift manifests across multiple dimensions with distinct timescales. Amplitude decay, where recorded neural potentials progressively diminish, typically emerges over weeks to months as encapsulation thickens and impedance rises. Chronically implanted arrays often lose 30%-50% of baseline amplitude within 6 to 12 months [15]. Waveform shape changes, reflecting shifts in the spatial relationship between electrodes and active neurons due to micromotion and local tissue remodeling, occur progressively over weeks to years and complicate unit isolation [15]. Unit loss, where previously isolated single neurons can no longer be distinguished from background activity, typically accelerates after several months and becomes pronounced by year one. These three mechanisms (amplitude decay, waveform distortion, and unit loss) are driven by distinct but overlapping biological processes: impedance increase from glial encapsulation, local inflammation causing neural death or drift, and geometric changes from tissue remodeling [15]. The cumulative effect is that a decoder calibrated at implantation may retain only 60%-70% of its initial accuracy after one year of chronic use and less thereafter [15]. This severity and timescale underscore why adaptive recalibration within closed-loop operation is not a convenience but a clinical requirement, and why control architectures must be designed from the outset to detect and compensate for these specific, predictable forms of drift.

Implantation into brain tissue initiates an inflammatory response that unfolds across distinct temporal phases and involves specific molecular cascades. In the acute phase (hours to days), local tissue damage triggers the release of damage-associated molecular patterns (DAMPs) and activation of the complement cascade, generating C3a and C5a that recruit peripheral immune cells. Resident microglia become activated and release pro-inflammatory cytokines, including interleukin-1 beta (IL-1β), tumor necrosis factor-alpha (TNF-α), and interleukin-6 (IL-6), which amplify local inflammation and promote blood-brain barrier permeability [21]. Concurrently, reactive oxygen species (ROS) accumulate from activated microglia and peripheral macrophages, causing oxidative stress that damages neuronal membranes and myelin. Over the chronic phase (weeks to years), this acute response does not simply resolve but transitions into a sustained, encapsulation-driven state [21]. Reactive astrocytes proliferate and form a dense glial scar around the implant, while microglia maintain a chronically activated phenotype, continuously producing low-level inflammatory mediators [21]. This persistent neuroinflammation is closely tied to rising tissue-electrode impedance and deterioration in signal quality as the encapsulating sheath progressively insulates the device from nearby neurons [21]. Understanding these specific molecular pathways and their temporal dynamics provides a mechanistic rationale for material interventions such as anti-inflammatory surface coatings, immunomodulatory polymer designs, and interface geometries that reduce mechanical irritation and dampen the glial response.

Scaling From Peripheral to Brain-Level Systems

Moreover, scaling closed-loop neuroprosthetics from peripheral nerves to the brain creates a harder control problem because cortical activity is distributed across dense, overlapping populations whose meaning shifts with context, internal state, and ongoing network dynamics rather than corresponding cleanly to a single movement or sensation. Peripheral interfaces often engage comparatively structured pathways with clearer functional organization, whereas brain-level systems must parse high-dimensional signals in which intention, attention, prediction, and error processing can coexist within the same brief interval. Decoding therefore becomes less a matter of reading a stable command channel than of detecting a changing brain state quickly enough to guide intervention, a requirement that has driven low-latency machine-learning approaches for brain-state detection in closed-loop sensing systems [14]. The implementation challenge is equally practical, because useful control depends on artifact-resistant recording, embedded processing, and stimulation that can operate over long periods of time rather than only in short demonstrations [22]. At the brain level, then, signal richness is inseparable from timing pressure and long-term systems integration.

Adaptive Control as Co-adaptation

Adaptive control, as employed in closed-loop operation in neuroprosthetic devices, also communicates with the user and makes individual learning demands. It is less a series of technical adjustments and more an exercise in co-adaptation. This is particularly true in a neuroprosthetic setting, where the user and algorithm learn from each other and adapt their respective control outputs over continued use [6]. To ensure drift is accounted for and control remains accurate, it is necessary for the system to adapt and learn from the user as the user strengthens neural learning in the given task. Adaptive design with continual updates in neuroprosthetic devices communicates with the user and allows learning based on the drift that is inevitable in the interface, electrodes, and muscles.

When security, reliability, and robustness become the target of rapid implantable bioelectronic system development efforts, it is not just with regard to their immediate performance characteristics. Technical usability in the clinical realm over time encompasses far more than just the success of surgical operation, accurate signal recovery, and processing in the acute phase or even through the unit's testbed lifespan. Continuous maintenance, performance decay, and frequent recalibration may be acceptable for short-term proof-of-concept demonstrations. However, these burdens severely limit clinical adoption, restricting deployment to specialized centers and preventing routine patient access to implantable neuroprosthetics. As implanted bioelectronic systems move closer to widespread clinical use, patient safety and device reliability have become central concerns in chronic neuromodulation and neuroprosthetic research. This is not a point to be appended as an afterthought upon proof of concept [8]. The same principle should apply to interface designs. The mechanisms driving long-term tissue harm and signal drift should be mitigated wherever possible. The device architecture offering the greatest success at this interface is that which supports long-lasting, stable recordings permitting accurate decoding on a closed-loop basis [9]. Broad access depends on implant architectures that deliver robustness, longevity, and recording stability. Equally important, these devices must be manageable within routine clinical workflows, not restricted to a small number of highly specialized centers. Broad clinical adoption requires implants designed for long-term durability from the outset. Currently, the difficulty of maintaining stable, safe performance in vivo restricts these devices to a small number of highly specialized centers, limiting patient access. Durable device design is therefore not merely an engineering preference but a prerequisite for routine clinical deployment.

The enduring themes of chronicity also increasingly include implant support strategies that prioritize comfort and stability to host tissue in the long term versus a rigid, wired construct that is more likely to stress tissue. Flexible substrates, soft encapsulation materials, and surface interfaces with better mechanical compatibility with neural tissue hold promise to reduce the risk of irritation from micromotion, preserve coupling through the remodeling of the implant in the host, and so on. In addition to supporting chronic survival, it stands the case that the combined sensing and stimulation architectures of a single implanted system promise unique benefits of simultaneous observation of interface drift and compensatory control modulation in the closed-loop device [13]. Full implantation is synonymous with this effort, the more so because it eliminates percutaneous leads and external hardware elements that might limit mobility but that also expose both the patient and the device to infection and chronic mechanical challenges. Materials and geometries that match neural tissue mechanics are critical to chronicity. When implants conform to tissue properties and movement, integrated closed-loop systems can seamlessly monitor and adjust stimulation in response to biological changes, blending therapeutic function into daily life without requiring frequent recalibration or intervention [8]. For all implantable bioelectronic systems, long-term clinical success ultimately depends on devices that remain mechanically stable, electrically functional, and biocompatible within host tissue over years of continuous use.

Finally, the most credible path toward durable brain-level neuroprosthetics lies in treating materials design and adaptive control as parallel responses to chronic failure. Recoverable conductors and deformable implant structures can reduce crack formation, preserve electrical continuity, and blunt the abrupt hardware losses that accumulate under long-term mechanical stress. But physical survival alone does not guarantee usable control, because neural interfaces also drift as signals change with context, fatigue, learning, and the evolving tissue-device relationship. For that reason, reliable chronic systems need closed-loop strategies that can detect background drift and recalibrate within operation rather than waiting for failure to become obvious [23]. Work on performance-recoverable closed-loop neuroprosthetic systems further underscores that stabilizing control under drift is not a secondary refinement but a core condition for long-term reliability [11]. Durable restoration, then, will depend on co-designed platforms in which material recovery and algorithmic adaptation jointly preserve function over time.

Looking ahead, closed-loop neuroprosthetic research is likely to shift from short proof-of-concept studies toward platforms designed for chronic, everyday use at the level of brain-based restoration. Since long-term recordings are vulnerable to drift and other instabilities that require adaptive closed-loop strategies to preserve useful control, a central priority will be maintaining durable neural sensing over years rather than over sessions [15]. That need makes continuous decoder updating and bidirectional feedback especially important, because stable performance will depend on systems that can adjust to changing signals while still returning meaningful sensory or task-relevant information to the user. Progress will also hinge on device architectures that support reliable long-term recordings and low-latency control as part of the same therapeutic loop, rather than treating hardware stability and decoding accuracy as separate problems [9]. If those elements are co-designed across materials science, embedded computation, and clinical workflow, brain-level neuroprostheses may become durable restorative systems for movement, sensation, and communication outside the laboratory.

## Conclusions

Lasting brain-level neuroprosthetic restoration will depend not on any single breakthrough but on fundamentally integrated development where materials science and adaptive control architecture advance in parallel, each constrained and enabled by the other. Self-healing interfaces that reduce mechanical mismatch only fulfill their clinical potential when paired with adaptive decoders that manage residual drift. Conversely, the most sophisticated algorithms cannot overcome hardware failures driven by material incompatibility. This co-designed approach departs from the historical sequential model in which hardware development precedes software tuning, and it reflects the reality that chronic restoration is a systems problem demanding integrated solutions.
